# Fatigue-induced Orosomucoid 1 Acts on C-C Chemokine Receptor Type 5 to Enhance Muscle Endurance

**DOI:** 10.1038/srep18839

**Published:** 2016-01-07

**Authors:** Hong Lei, Yang Sun, Zhumin Luo, Gregory Yourek, Huan Gui, Yili Yang, Ding-Feng Su, Xia Liu

**Affiliations:** 1 Department of Pharmacology, School of Pharmacy, Second Military Medical University, Shanghai, 200433, China; 2 DL Biotech USA, Gaithersburg, MD, USA; 3 Laboratory of Translational Medicine, Suzhou Institute of Systems Medicine, Center for Systems Medicine, Chinese Academy of Medical Sciences, Suzhou 215123, Jiangsu, China

## Abstract

Understanding and managing fatigue is a significant challenge in clinic and society. In attempting to explore how the body responds to and regulates fatigue, we found in rodent fatigue models that orosomucoid 1 (ORM1) was significantly increased in multiple tissues, including blood and muscle. Interestingly, administration of exogenous ORM1 increased muscle glycogen and enhanced muscle endurance, whereas ORM1 deficiency resulted in a significant decrease of muscle endurance both *in vivo* and *in vitro*, which could largely be restored by exogenous ORM1. Further studies demonstrated that ORM1 can bind to C-C chemokine receptor type 5 (CCR5) on muscle cells and deletion of the receptor abolished the effect of ORM1. Thus, fatigue upregulates the level of ORM1, which in turn functions as an anti-fatigue protein to enhance muscle endurance via the CCR5 pathway. Modulation of the level of ORM1 and CCR5 signaling could be a novel strategy for the management of fatigue.

Fatigue is a commonly experienced phenomenon with important consequences both in daily activities, exercise, athletic competition, and under many pathological conditions. Although there are a variety of definitions for fatigue, it usually manifests as a reversible decline in performance for affected individuals, which was commonly utilized as a measurement for fatigue in animal models^1^,^2^. Among the various forms of fatigue, physical fatigue (also referred as peripheral fatigue) is the experience of exhaustion following strenuous physical effort, whereas central or mental fatigue is largely the subjective self-reported feeling of fatigue, which is frequently associated with other psycho-social factors, such as sleep disorder, depression, and anxiety^3^,^4^. While physical fatigue is usually accompanied by fuel depletion and lactic acid accumulation, there are seldom metabolic changes during central fatigue except when exercise is involved. Noteworthily, both peripheral and central fatigue could induce stress response and acute phase reaction^5^,^6^. It has been shown that there is a close association between the level of plasma C-reactive protein and various types of fatigue^7^,^8^. The terms acute fatigue and chronic fatigue are also used under certain circumstances. It is generally believed that acute fatigue is a protective mechanism and often relieved by rest or life-style change. In contrast, chronic fatigue is considered pathological, lasts at least 6 months, and negatively affects physical and mental function^3^,^4^. Among which, chronic fatigue syndrome is an extensively studied medical condition, whose cause and mechanism are still not well understood.

Fatigue represents a significant personal, social, economic, and medical challenge. A number of surveys have found that up to half of the general population experienced chronic fatigue sometimes in their life, and at least 20% of the patients with fatigue seek medical care^9^,^10^,^11^. In clinics, fatigue is often associated with various diseases, particularly cancer, inflammatory, and autoimmunity diseases, such as multiple sclerosis and systemic lupus erythematous^12^,^13^. At present, there are still no specific factors that have been consistently associated with a particular type of fatigue, and there are no official or semi-official recommendations for the treatment of various forms of fatigue^10^,^14^,^15^. Nonspecific treatments, such as nutritional supplements, immunological agents (i.e. IgG and interferon α2), and pharmacological products (i.e. steroids, modafinil and caffeine) have been used experimentally, which usually have negative, inconclusive or minor effects in various studies^16^,^17^,^18^,^19^.

Although fatigue has been recognized and studied for centuries, its etiology remains a matter of considerable debate. Multiple factors, including viral infections, immune system abnormalities, psychological traits, hypothalamic-pituitary-adrenal dysfunction, oxidative stress, and energy depletion could induce or contribute to the development of fatigue^3^,^10^. How these factors interact with the nerve and muscle systems and cause the onset of fatigue remain the focuses of on-going investigations. It is evident that many of them may exert their influence on multiple organ systems directly and/or indirectly through humoral factors, such as cytokines and hormones^20^.

We hypothesized that, similar to stress, fatigue may induce a general response in affected individuals. In attempting to characterize the response, we used proteomics methods to compare liver protein expressions in fatigued rodents with that of controls. It was found that the expression of acute phase protein ORM1 is increased by various forms of fatigue. Interestingly, ORM1 is able to act on cell membrane receptor CCR5 to increase muscle glycogen and enhanced muscle endurance, forming a positive feedback to exert anti-fatigue function. Therefore, ORM1 and CCR5 are regulators of fatigue and could be novel targets for the therapeutic intervention of fatigue.

## Results

### Orosomucoid 1 (ORM1) expression is significantly increased in fatigued rats

**Figure 1:**
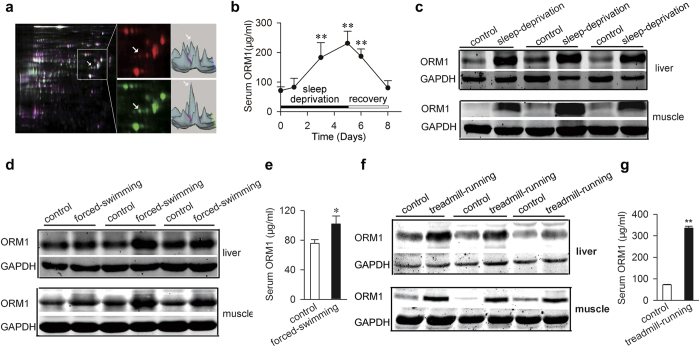
Orosomucoid 1 (ORM1) is significantly elevated in fatigued rats. (**a**) Left: 2D difference gel electrophoreses (DIGE) comparison of liver proteins derived from control (Cy5, red) and sleep-deprived (Cy3, green) fatigue rats. Right: the corresponding 3-D views of fluorescence intensity. The white arrow heads indicate ORM1. (**b**) ORM1 levels in sera from rats after sleep deprivation were determined by ELISA (n = 7 mice per time point). (**c**) Representative western blots of ORM1 in muscle and liver tissues from control and sleep-deprivation rat (n = 6). (**d**,**e**) ORM1 expression in muscle, liver tissues (**d**), or serum (**e**) in control and forced swimming fatigue rats (n = 6). (**f**,**g**) ORM1 expression in muscle, liver tissues (**f**), or serum (**g**) in control and treadmill running rats (n = 6). All data are expressed as the mean ± s.d. *P < 0.05, **P < 0.01 by Dunnett’s t test.

Fatigue can be induced by a variety of factors through many different mechanisms, which represent a significant challenge for experimental mechanistic studies. We sought to understand fatigue from a different direction and hypothesized that there is a general response to fatigue in affected individuals. A rat sleep deprivation model of fatigue was utilized to get insight of the response^2^,^21^. After a 5-day sleep deprivation, the rats had significant reduced muscle and liver glycogen, and increased blood lactate, indicatives of fatigue (Supplementary Fig. 1). To find fatigue-induced alterations of protein expression in the liver of these rats, differentially expressed spots on the 2D- electrophoresis gels were analyzed by using mass spectrometry. We identified seven proteins (Supplementary Table 1) whose expressions were substantially changed in response to fatigue. Among which, ORM1, also known as α1 acid glycoprotein, is the most significantly increased protein (Fig. 1a and Supplementary Fig. 2). As a major acute-phase protein, ORM1 is mainly synthesized and secreted by the liver^22^. Its level in rat serum was then measured over a 5-day fatigue paradigm and a 3-day recovery period. As shown in Fig. 1b, the level of ORM1 increased gradually as the fatigue time prolonged, reached the highest level at Day5, and decreased gradually during the recovery period, indicating that it is associated with the severity of fatigue. It is worth noting that many extra-hepatic tissues and cells are also capable of producing ORM1 under various experimental conditions^23^,^24^. We therefore examined the expression of ORM1 by immunoblotting in a number of tissues and found that, in addition to the liver, ORM1 is significantly increased in the skeletal muscle (Fig. 1c) and the heart from fatigued rats (data not shown). ORM1 upregulation is not limited to fatigue induced by sleep deprivation. Weight-loaded forced swimming and treadmill running, two commonly used physical fatigue models (Supplementary Fig. 3 and Supplementary Fig. 4)^25^, also resulted in significant increase of ORM1 expression in rat muscle, liver, and serum (Fig. 1d–g).

### ORM1 is an endogenous anti-fatigue protein

**Figure 2:**
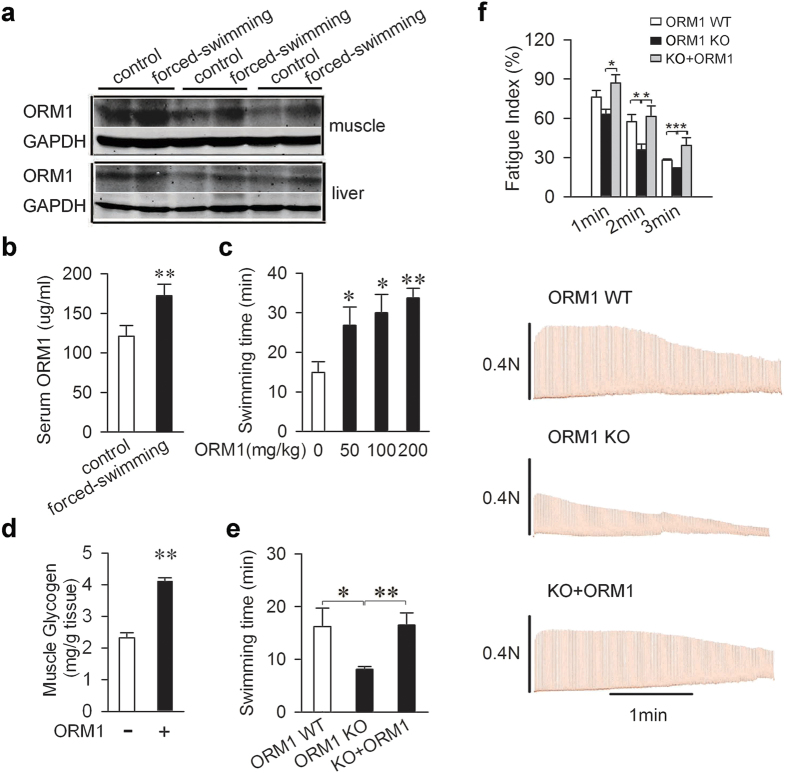
ORM1 is an endogenous anti-fatigue protein. (**a**,**b**) ORM1 expression in muscle, liver tissues (**a**), or serum (**b**) in control and forced swimming mice (n = 6). (**c**) Effect of ORM1 on mice swimming ability. Swimming tests were carried out 30 minutes after the mice were intravenously injected with exogenous ORM1 or vehicle (n = 7 per group). (**d**) Muscle glycogen contents 30 min after treatment with vehicle or 200 mg/kg ORM1 in mice (n = 7 per group). (**e**) Swimming times of wild-type mice (WT), ORM1 knockout mice (KO), and ORM1 knockout mice injected with 200 mg/kg of ORM1 (n = 6 per group). (**f**) Representative records of electrically evoked contractions of soleus muscle isolated from C57/BL mice treated as mentioned in (**e**) (n = 6). All data are expressed as the mean ± s.d. *P < 0.05, **P < 0.01 by Dunnett’s t test.

To assess the potential role of ORM1 in fatigue, we selected the mice weight-loaded forced swimming test as a model for practical reasons (i.e. availability of genetic modified mice, affordability of purified ORM1, and experimental cycle)^25^. As shown in Fig. 2a,b, forced swimming also led to increased ORM1 in the liver, muscle, and serum of mice. Purified ORM1 was then given intravenously to mice before they were forced to swim, and it increased swimming times dose-dependently (Fig. 2c and Supplementary Fig. 5). While fatigue is associated with glycogen depletion (Supplementary Figs 1b, 3b, and 4b)^26^, administration of ORM1 also significantly increased muscle glycogen content (Fig. 2d).

To further determine its role in fatigue, ORM1-deficient mice were generated through targeted deletion of exon 1–5 of the *ORM1* gene (Supplementary Fig. 6). The mice were born with Mendelian rations and appeared develop normally. Compared with their littermates, the ORM1-deficient mice could only swim about half of the length of time (Fig. 2e). Furthermore, administration of purified ORM1 to these mice restored the swimming time completely (Fig. 2e). To further clarify whether ORM1 can directly affect the endurance of skeletal muscles, isolated mouse soleus muscle was utilized to electronically induce fatigue *in vitro*. As shown in Fig. 2f, ORM1 knockout significantly decreased the fatigue index at 2 and 3 minutes of the stimulation, and administration of purified ORM1 to these mice restored, even increased, the fatigue index, suggesting that the ORM1 is an endogenous anti-fatigue protein that may directly enhance muscle endurance.

### ORM1 specifically binds to the C-C chemokine receptor type 5 (CCR5) in skeletal muscle

**Figure 3:**
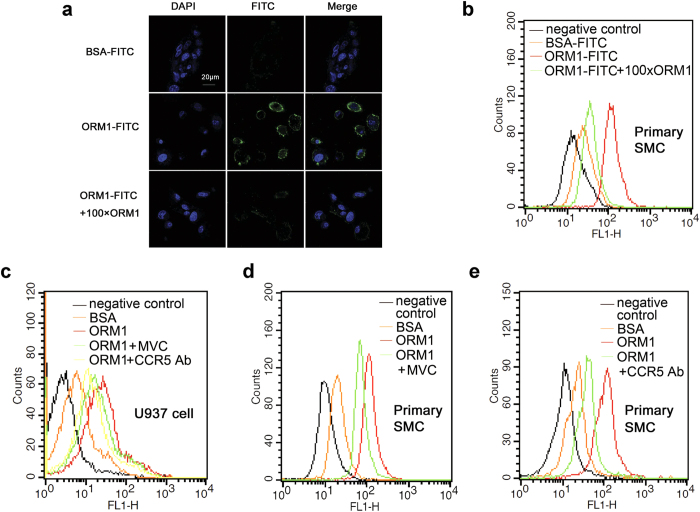
ORM1 specifically binds to the C-C Chemokine receptor type 5 (CCR5) in skeletal muscle. (**a**) Representative immunofluorescence images of mouse primary skeletal muscle cells stained for FITC labelled BSA, FITC labelled ORM1 in absence or presence of 100 folds of non-FITC labelled ORM1 (n = 3). Nuclei were stained with DAPI (blue). (**b**) Representative flow cytometry analysis results of mouse primary skeletal muscle cells incubated as indicated in (**a**), and cells stained with blank were used as negative control (n = 3). (**c**) Representative histogram of U937 cells incubated with blank (negative control), FITC-labeled BSA, FITC-labeled ORM1 in the absence or presence of CCR5 antagonist Maraviroc or anti-CCR5 antibody. (**d**,**e**) Representative histogram of mouse primary skeletal muscle cells stained with blank (negative control), FITC-labeled BSA, FITC-labeled ORM1 in the absence or presence of Maraviroc (**d**) or anti-CCR5 antibody (**e**). Maraviroc: MVC. Skeletal muscle cell: SMC.

Many different activities, including regulating angiogenesis, modulating inflammatory and immune response, have been attributed to ORM1^22^,^27^. A number of studies found that it could act on multiple immune cells, such as neutrophils and macrophages, although the receptors and signal transduction pathways for these actions are not well understood^28^. Since ORM1 increases muscle glycogen content and enhances muscle endurance, we asked whether it could recognize receptor on muscle cells. ORM1 was labelled with fluorescein FITC and incubated with the mouse primary skeletal muscle cells (Supplementary Fig. 7). As shown in Fig. 3a, FITC-ORM1 bound to the skeletal muscle cell membrane, and the binding could be inhibited completely by excessive unlabeled ORM1. This was further confirmed with Flow cytometry analysis, which showed that all skeletal muscle cells bind specifically with ORM1 (Fig. 3b). It has been reported that ORM1 binds to macrophages cytokine receptor CCR5^29^, a G-protein-coupled receptors that is also expressed on myoblast C2C12 cells^30^. While known as a receptor involved in inflammation and immune response, CCR5 participates the injury responses of skeletal, myocardial, and smooth muscles^30^,^31^,^32^. We therefore examined whether ORM1 could bind to CCR5 to exert its effects. Use was made of the monocyte cell line U937 cells that express functional CCR5. Flow cytometry analysis showed that FITC-labeled ORM1 bound toU937 cells, and the binding was inhibited significantly by an anti-CCR5 antibody and by the CCR5 antagonist Maraviroc (Fig. 3c). Similarly, the binding of ORM1 to the primary skeletal muscle cells was inhibited partially by Maraviroc and the anti-CCR5 antibody (Fig. 3d,e), indicating that ORM1 can act on CCR5 of muscle cells.

### CCR5 in skeletal muscle mediates the anti-fatigue effect of ORM1

**Figure 4:**
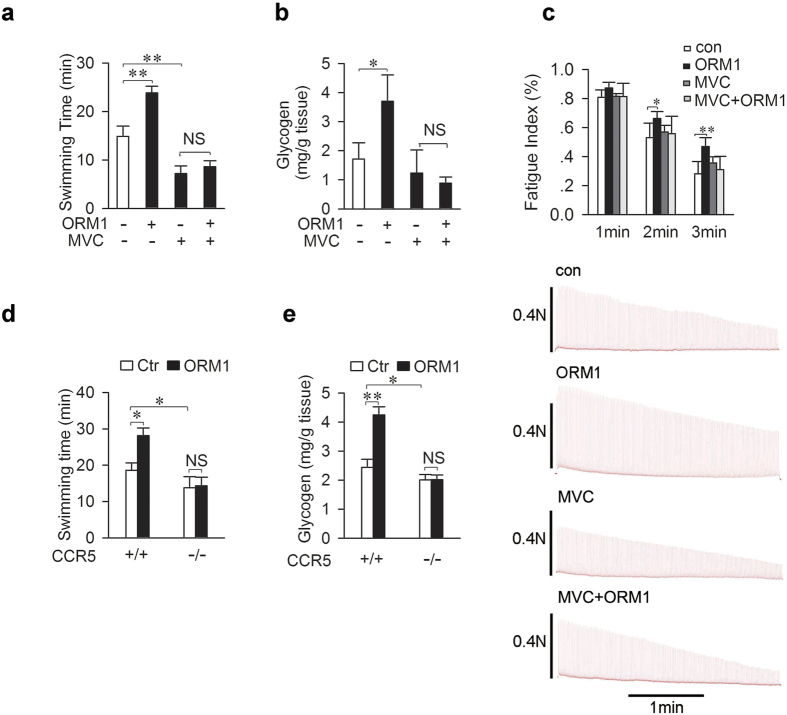
CCR5 in skeletal muscle mediates the anti-fatigue effect of ORM1. (**a**,**b**) Swimming time ((**a**) n = 7) and muscle glycogen content ((**b**) n = 4) were assessed 30 minutes after intravenous injection of ORM1 (200 mg/kg) or vehicle in the presence or absence of Maraviroc (200 mg/kg, via gastric gavage, for three days). (**c**) Representative records of electrically evoked contractions of soleus muscle isolated from C57/BL mice treated as mentioned in (**a**) (n = 6). (**d**,**e**) Swimming times (**d**) and muscle glycogen content (**e**) were determined 30 min after treatment with ORM1 (200 mg/kg) or vehicle (Ctr) in CCR5^+/+^ or CCR5^−/−^ mice (n = 4–6 per group). Maraviroc: MVC. Data are expressed as the mean ± s.d. NS, not significant, *P < 0.05, **P < 0.01 by Dunnett’s test.

These results prompted us to examine whether CCR5 is involved in the effects of ORM1 on glycogen level and fatigue. As shown in Fig. 4a,b, intravenous injection of CCR5 antagonist Maraviroc abolished the stimulatory effect of ORM1 on swimming ability and muscle glycogen content. However, Maraviroc alone also decreased swimming times significantly (Supplementary Fig. 8), suggesting that endogenous ORM1 might exert its anti-fatigue action via CCR5. Alternatively, the antagonist could have off-target effects.

To determine whether ORM1 can act directly on skeletal muscles via CCR5, isolated mouse soleus muscle was utilized to electronically induce fatigue *in vitro*. As shown in Fig. 4c, ORM1 increased significantly the fatigue index at 2 and 3 minutes of the stimulation. The increases were prevented by Maraviroc, which did not affect the index by itself. These results indicated that ORM1 could act on muscle CCR5 to enhance muscle function. CCR5-deficient mice were further used to explore its role in the anti-fatigue action of ORM1. These mice had a small but significant decrease in swimming time and muscle glycogen content as compared to controls (Fig. 4d,e). Furthermore, administration of ORM1 failed to increase swimming time and did not increase muscle glycogen content in these CCR5-deficient mice, demonstrating that the receptor is required for its anti-fatigue action.

## Discussion

Fatigue is a commonly experienced, but non-specific and highly subjective symptom. Detecting and evaluating fatigue is a common task both in various society activities and in clinical practice. While physical or acute fatigue can often be identified or characterized through functional and metabolic changes, diagnosis of mental or chronic fatigue usually depends on subjective self-reported feeling^3^,^4^. Therefore, understanding its biological mechanisms and discovering associated characteristic biomarkers are essential for improving the diagnosis and treatment of mental or chronic fatigue. We found in this study that ORM1 is significantly elevated in response to various forms of fatigue, indicating that the increased level of ORM1 is an indicative of fatigue and might be particularly informative for the diagnosis and management of mental fatigue. Furthermore, the fatigue-induced ORM1 has substantial effects on muscle endurance, representing a positive feedback mechanism to resist fatigue and maintain homeostasis. Therefore, modulation of the ORM1/CCR5 system could be a novel strategy for therapeutic intervention of fatigue.

As an acute phase protein, the serum level of ORM1 can elevate to 2–10 folds under many pathological conditions, such as sepsis, tumor, tissue injury^33^. It has been shown that glucocorticoid hormone is an important regulator of *ORM1* expression in liver cells and a glucocorticoid responsive element (GRE) has been identified in close proximity to the starting site of its transcription^34^. We found in the present study that the serum corticosterone levels were also significantly increased in fatigue rodents, indicating that it may be responsible for the increase of ORM1. Noteworthily, it has been shown that nuclear bile acid receptor farnesoid X receptor regulates hepatic ORM1 expression similar to that of glucocorticoid receptor^35^. Additionally, cytokines such as IL-1, TNF-α, and IL-6 are also able to increase ORM1^33^,^36^. It is conceivable that these cytokines and transcription factors may also contribute to the elevation of ORM1 level in body’s reaction to fatigue.

CCR5 belongs to the superfamily of G-protein-coupled receptors, which contain seven transmembrane helices and transmit signals from extracellular signals to intracellular pathways through heterotrimeric G-proteins. Multiple chemokines, including MIP-1a, MIP-1b, RANTES, and MCP-2, can function as CCR5 ligands. In addition to its roles in inflammation and immune response, CCR5 also functions as a coreceptor for HIV to enter into cells, leading to the development of many small molecular inhibitors of the receptor. While MIP-1a and RANTES can recognize CCR5 and other chemokine receptors, ORM1 binds to CCR5 as well as a number of other receptors^28^,^37^. It has been reported that both CCR5 and ORM glycans are involved in the binding of ORM1 to macrophages^29^. In our studies, the incomplete inhibition of ORM1 binding by the CCR5 antibody and antagonist in U937 or C2C12 cells is likely due to the ability of the heavily glycosylated ORM1 to bind to other cell surface proteins. Noteworthily, the anti-fatigue action of ORM1 effects is completely abolished by CCR5 antagonist or knockout, indicating that CCR5 is the major mediator of the effect.

Many different activities, including modulating immunity, binding and carrying drugs, maintaining the barrier function of capillary, and mediating the sphingolipid metabolism^33^,^38^,^39^, have been attributed to ORM1. We found in this study that ORM1 increases muscle glycogen, whose level has been demonstrated in a variety of studies closely related to the endurance of skeletal muscles^26^,^40^. It has also been reported that ORM1 increased glucose uptake in 3T3-L1 adipocytes^41^, and CCR5 activation enhanced glucose uptake in activated T cells^42^, indicating that ORM1 may activate CCR5 and increase glucose uptake to elevate glycogen content in muscle cells. Interestingly, one of the major side effects of the CCR5 antagonist in the treatment of HIV infection is fatigue^43^. It is of great interesting to further examine whether it is related to blocking ORM1 action and whether administration of ORM1 could relieve this side effect. It is also worth noting that these responses in present study were found in rodent fatigue models. Whether ORM1 possesses similar activity in human remains to be further investigated.

## Materials and Methods

### Reagents

ORM1 was purchased from Sigma (St. Louis, MO). BSA was obtained from Boguang Biological Technology (Shanghai). FITC-labeled ORM1 and BSA were made by Youke Biological Technology (Shanghai). CCR5 antibody was purchased from LifeSpan Biosciences (Seattle, WA). CCR5 antagonist Maraviroc was generously provided by Professor XinXie (Shanghai Institute of Material Medica, CAS). Antibodies specific to ORM1 and GAPDH were obtained from Cell Signaling (Danvers, MA).

### Animals

C57BL/6 mice (18–22 g) and Sprague-Dawley rats (180–200 g) were purchased from Sino-British SIPPR/BK Laboratory Animals (Shanghai, China). The CCR5-deficient mice (B6.129P2-Ccr5^tm1Kuz^/J, Stock Number: 005427) were obtained from Jackson Laboratory (Bar Harbor, MA). Mice were 6 to 8 weeks of age at the start of the experiments. All animals were maintained at 22 °C on a 12-hour light/dark cycle with free access to water and a standard rodent diet. Animal experimentation: all animal experiments were performed in strict accordance with the National Institute of Health’s “Guide for the Care and Use of Laboratory Animals”, with the approval of the Scientific Investigation Board of the Second Military Medical University (No. SMMU-SIB-201303092). In the following experiments, all animals were euthanized by cervical vertebra dislocation.

### Sleep-deprivation fatigue

Male Sprague-Dawley rats were deprived of sleep for 5 consecutive days in a cage filled with water (23 ± 1 °C) to a height of 1.5 cm as described previously^2^.

### Weight-loaded forced swimming

Mice or Rats were placed individually in a cylindrical glass tank (46 cm tall, 20 cm in diameter) of water at 21–23 °C. A load consisting of a steel ring weighing 8% of each body weight was attached to the proximal end of the tail. The swimming time was measured from when the rat or mouse began swimming to when it could not return to the surface of the water 10 s after sinking^44^. This experiment was performed double-blinded.

### Treadmill running

Rats were subjected to run on a treadmill system at 0° slope to make them exhausted^45^. The speed began at 12 m/min, and was increased every 5 min at the increment of 3 m/min until reaching 30 m/min. During the one-week adaptation training, rats were forced to run an hour per day at the speed of 30 m/min. At the last day of the week, they were forced to run at the speed of 30 m/min until they were unable to keep pace with the treadmill for up to 10 s. Then they were removed from the treadmill and placed in a supine position to check if they were exhausted. If the rats could not right themselves, they were considered as exhausted, and counted the time. Otherwise, exercise was continued until exhaustion occurred.

### DIGE and mass spectrometry

Liver proteins were labeled with the CyDye DIGE fluor minimal labeling kit (GE Healthcare, Piscataway, NJ). For isoelectric focusing electrophoresis, the labeled samples were placed in a strip holder using a step gradient protocol (30 v for 6 hrs, 60 v for 6 hrs, 200 v for 1 hr, 500 v for 1 hr, 1000 v for 1 hr and 8000 v for 6 hrs). After equilibrated in SDS equilibration solution, proteins in the strips were separated on a 12.5% homogeneous SDS-PAGE gel. DIGE images were visualized with the Typhoon Trio variable imager (GE Healthcare) and analyzed using DeCyder software v6.5 (GE Healthcare, Piscataway, NJ). The ratios of the log-standardized protein spot abundances (differences in expression) between the groups were computed and analyzed with a one-way ANOVA. Differentially expressed protein spots were excised from the stained gel for identification with 4700 MALDI-TOF/TOF Proteomics Analyzer (Applied Biosystems, Foster City, CA, USA).

### Immunoblotting

Tissue samples were harvested after PBS perfusion to evacuate the circulating blood and lysed with the M-PER protein extraction reagent (Pierce, Rockford, IL) supplemented with a protease inhibitor mixture (Calbiochem, San Diego). After centrifugation, proteins in the supernatant were separated by SDS-PAGE and transferred onto PVDF membranes. The membranes were then probed for specific proteins. Equal loading of the samples was confirmed by re-probing the blots for GAPDH.

### ELISA

Serum levels of ORM1, CRP and corticosterone were detected by ELISA kit (Biotechnology Systems, Shanghai, China) according to the manufacture’s instruction.

### Blood glucose

Blood glucose was measured using glucose monitor and associated test strips (Life Scan, CA, USA).

### Lactate detection

Lactate was estimated using lactate oxidase method based kit (Randox, UK) according the manufacture’s instruction.

### Glycogen assay

Muscle and liver glycogen were extracted and determined with a glycogen assay kit (Glycogen assay kit, Biovision, USA).

### Generation and genotyping of ORM1 knockout mice

ORM1 knockout mice were generated by the laboratory of Shanghai Biomodel Organism Science & Technology Development (Shanghai) through construction of vector targeting exons 1 to 5 of ORM1 gene, transfection and selection of ES cells, and injection of ES cells into blastocysts of C57BL/6J mice. The resulted chimerical males were bred with female C57BL/6J mice to produce heterozygous mutant mice, which were then inter-crossed to obtain homozygous and wild-type (WT) mice. The ES cells were identified by PCR amplification and gene sequencing. The primers used were P1 (5′-GAGACGTGTGCATACTGTGATGAGC-3′), P2 (5′-CCTCCCCCGTGCCTTCCTTGAC-3′), P3 (5′-AACAGCATCAGGAGGCTCAAAATCA-3′) and P4 (5′-CTGAGCCCAGAAAGCGAAGGA-3′). For genotyping, mice tail DNA was amplified using the ABI PRISM 7700 Sequence Detection System (TaqMan). The primers for ORM 1 knockout mice were: P1: 5′-ACTGTCCCTCTATGGTAAGCACC-3′, P2: 5′-CTGAGCCCAGAAAGCGAAGGA-3′, P3: 5′-CACCCTGGCACCGGATATTCC-3′. (Wild type = 526 bp (P1/P3); Mutant = 598 bp (P2/P3)).

### Cell culture

U937 cells were purchased from FuMeng Gene Bio-technology (Shanghai) and maintained in RPMI 1640 medium supplemented with L-glutamine and 10% fetal calf serum. Mouse primary skeletal muscle cells were provided by iCell Bioscience (Shanghai, China) and cultured in complete DMEM medium containing 10% fetal bovine serum, 100 U/ml Penicillin/0.1 mg/ml Streptomycin at 37 °C and 5% CO_2_.

### Confocal microscopy

The mouse primary muscle cells on slides were incubated with FITC labelled BSA (control) or FITC-labelled ORM1 in the absence or presence of 100-fold excess of un-labelled ORM1 for 1 hour. After rinsed with PBS and fixed with 4% paraformaldehyde for 15 mins, they were washed with PBS and stained with DAPI for 2–5 mins. Images were taken using the confocal laser scanning microscope (FV1000, Olympus, Japan).

### Flow cytometry

The interaction of ORM1 with the surface of the mouse primary skeletal muscle cells or U937 cells was analyzed using flow cytometry. Briefly, cells were blocked with 5% BSA and incubated with blank (negative control), FITC-conjugated BSA, FITC-conjugated ORM1, or FITC-conjugated ORM1 in combination with CC5 antagonist Maraviroc or anti-CCR5 antibody on ice for 1 hour, washed with PBS three times, and then analyzed by flow cytometry.

### Fatigue induction in isolated muscle

Muscle fatigue was induced as previously reported^46^. Briefly, mice soleus muscle was isolated. One end of muscle was fixed, and another was linked to the sensor of a biotic signal collection and processing system (MedLab-U/4CS, MeiYi, Nanjing, China). The muscle was electrically evoked contractions by trains of stimuli at 10 V lasting 5 ms and delivered each second for 3 minutes. The ratio of tension at 1 minute, 2 minutes or 3 minutes to the initial tension (average of the first 5 contractions) was expressed as the fatigue index.

### Statistical analysis

SPSS version 19.0 was used for statistical analyses. All data were checked for normality using the Shapiro-Wilk test. Equality of variances was checked through the Levene’s Test. When all data followed a normal distribution and the variances were homogenous, they were analyzed using ONE WAY ANOVA followed by Dunnett’s test for multiple comparisons. Data are presented as means ± S.D. P < 0.05 were considered as statistically significant.

## Additional Information

**How to cite this article**: Lei, H. *et al.* Fatigue-induced Orosomucoid 1 Acts on C-C Chemokine Receptor Type 5 to Enhance Muscle Endurance. *Sci. Rep.* **6**, 18839; doi: 10.1038/srep18839 (2016).

## Supplementary Material

Supplementary Information
