# Supplementary material for: Fatigue-induced Orosomucoid 1 Acts on C-C Chemokine Receptor Type 5 to Enhance Muscle Endurance
Source: Sci Rep. 2016 Jan 7;6:18839. doi: 10.1038/srep18839 (PMC4703980; doi:10.1038/srep18839)
Supplement: Supplementary Information [file srep18839-s1.doc]

Supplementary Materials for

# Fatigue-induced Orosomucoid 1 Acts on C-C Chemokine Receptor Type 5 to Enhance Muscle Endurance

Hong Lei, Yang Sun, Zhu-Min Luo, Gregory Yourek, Huan Gui, Yili Yang, Ding-Feng Su, and Xia Liu^*^

*Correspondence authors. E-mail: lxflying@aliyun.com

**This PDF file includes:**

Fig.S1. Glycogen and serological changes in sleep deprivation rats.

Fig.S2. MALDI TOF/TOF MS analysis of ORM1.

Fig.S3. Glycogen and serological changes in forced swimming rats.

Fig.S4. Glycogen and serological changes in treadmill running rats.

Fig.S5. Serum ORM1 level after ORM1 intravenous injection.

Fig.S6. Generation and identification of ORM1 knockout mice.

Fig.S7. Identification of the mouse primary skeletal muscle cells.

Fig.S8. Maraviroc affected swimming time in mice.

Table. S1. Differentially expressed proteins in liver from sleep deprived rat.


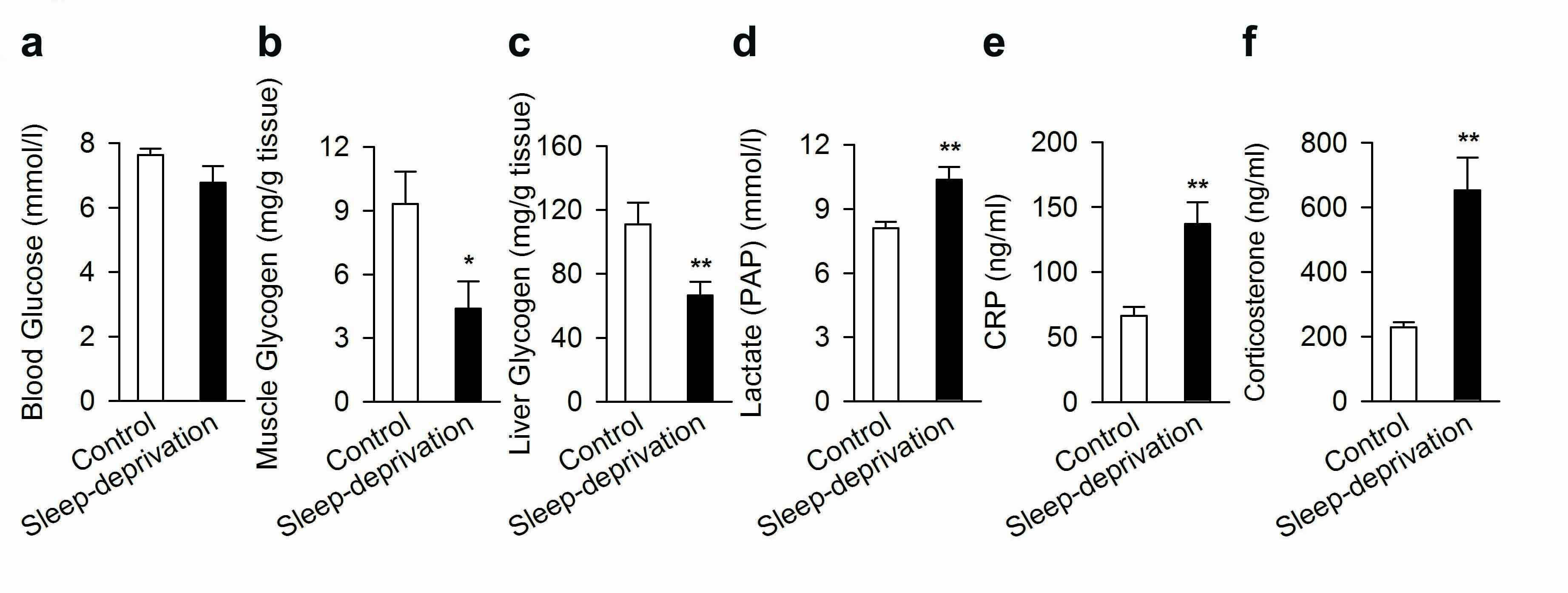


**Fig.S1**. Glycogen and serological changes in sleep deprivation rats. Blood glucose (**a**), muscle glycogen (**b**), liver glycogen (**c)**, serum lactate (**d**), serum CRP (**e**), and serum corticosterone (**f**) in control and sleep deprivation rats (n=6). Data are mean ± s.d.* P< 0.05, ** P< 0.01 by Dunnett’s test.


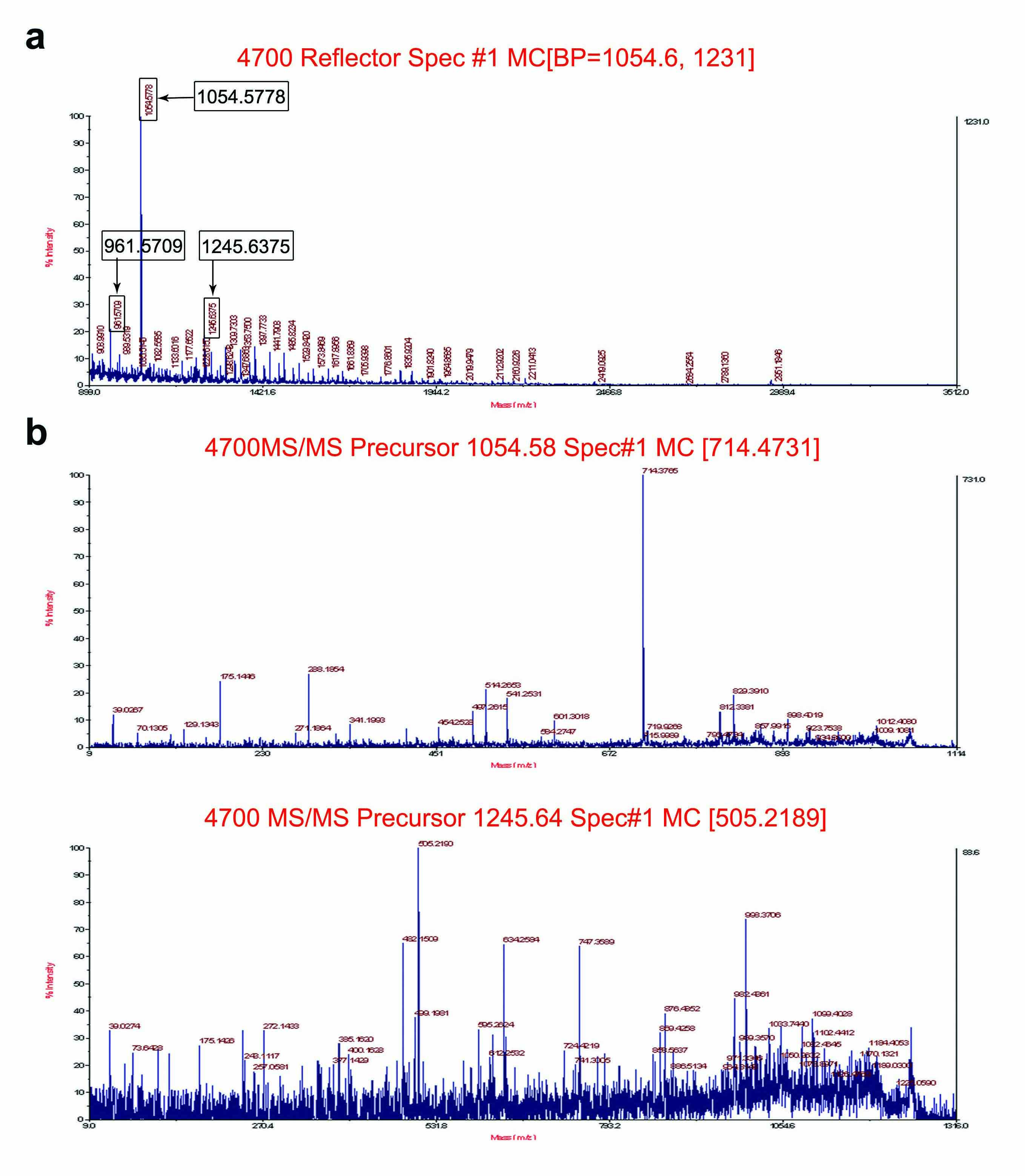


**Fig.S2**. MALDI TOF/TOF MS analysis of ORM1. (**a**) The peptide mass fingerprint of protein spot identified as ORM 1 based on the matched peaks (indicated with arrows). (**b**) MS signals were derived from the parent ion at *m*/*z* 1054.5778 and *m*/*z* 1245.64, for which the amino acid sequence, KPDLSPELR and QQLELEKETK were deduced based upon these b-ions and y-ions in tandem MS spectrum, respectively.


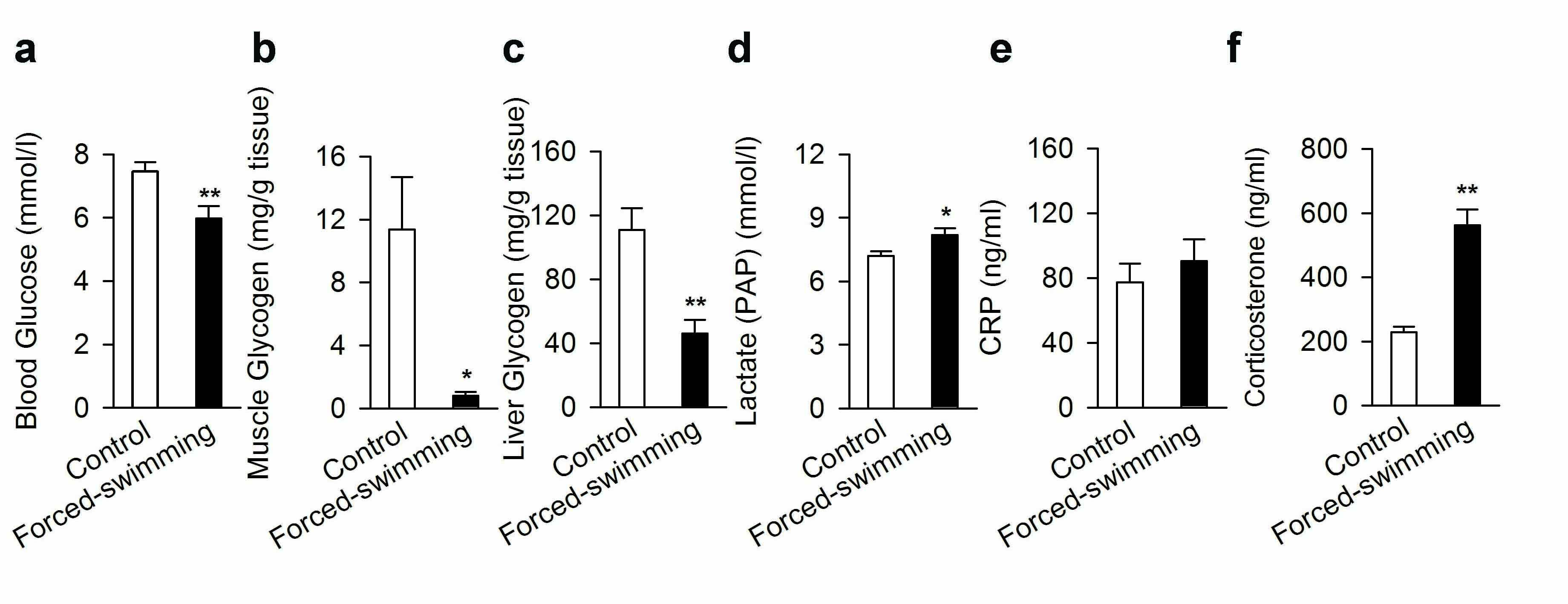


**Fig. S3.** Glycogen and serological changes in forced swimming rats. Blood glucose (**a**), muscle glycogen (**b**), liver glycogen (**c**), serum lactate (**d**), serum CRP (**e**), and serum corticosterone (**f**) in control and forced swimming rats (n=6). Data are mean ± s.d.* P< 0.05, ** P< 0.01 by Dunnett’s test.


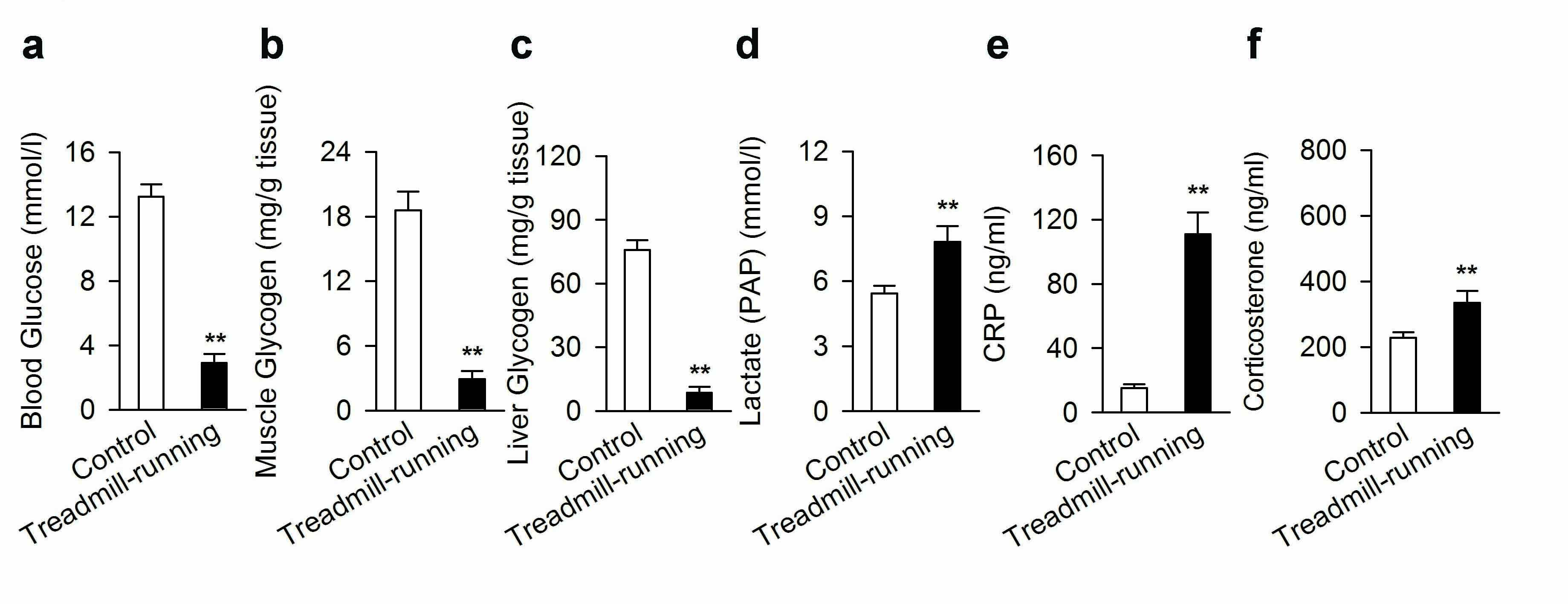


**Fig. S4.** Glycogen and serological changes in treadmill running rats. Blood glucose (**a**), muscle glycogen (**b**), liver glycogen (**c**), serum lactate (**d**), serum CRP (**e**), and serum corticosterone (**f**) in control and treadmill running rats (n=6). Data are mean ± s.d. * P< 0.05, ** P< 0.01 by Dunnett’s test.

**
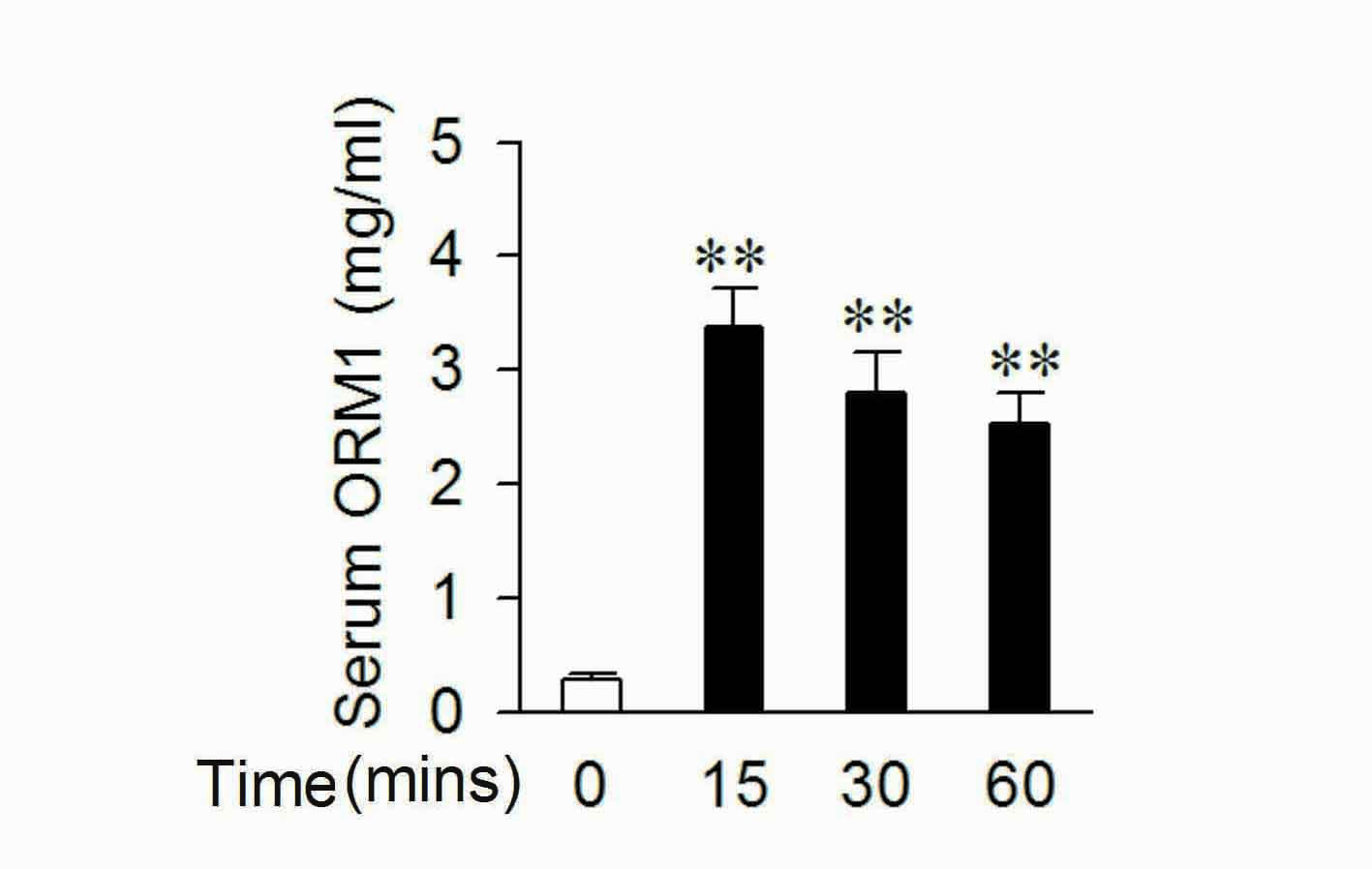
**

**Fig.S5.** Serum ORM1 level after ORM1 intravenous injection. ELISA detection of serum ORM1 at the time indicated in mice after tail intravenous injection with vehicle or 200 mg/kg ORM1. n=7 mice pre group. Data are mean ± s.d. ** P< 0.01 by Dunnett’s test.

**
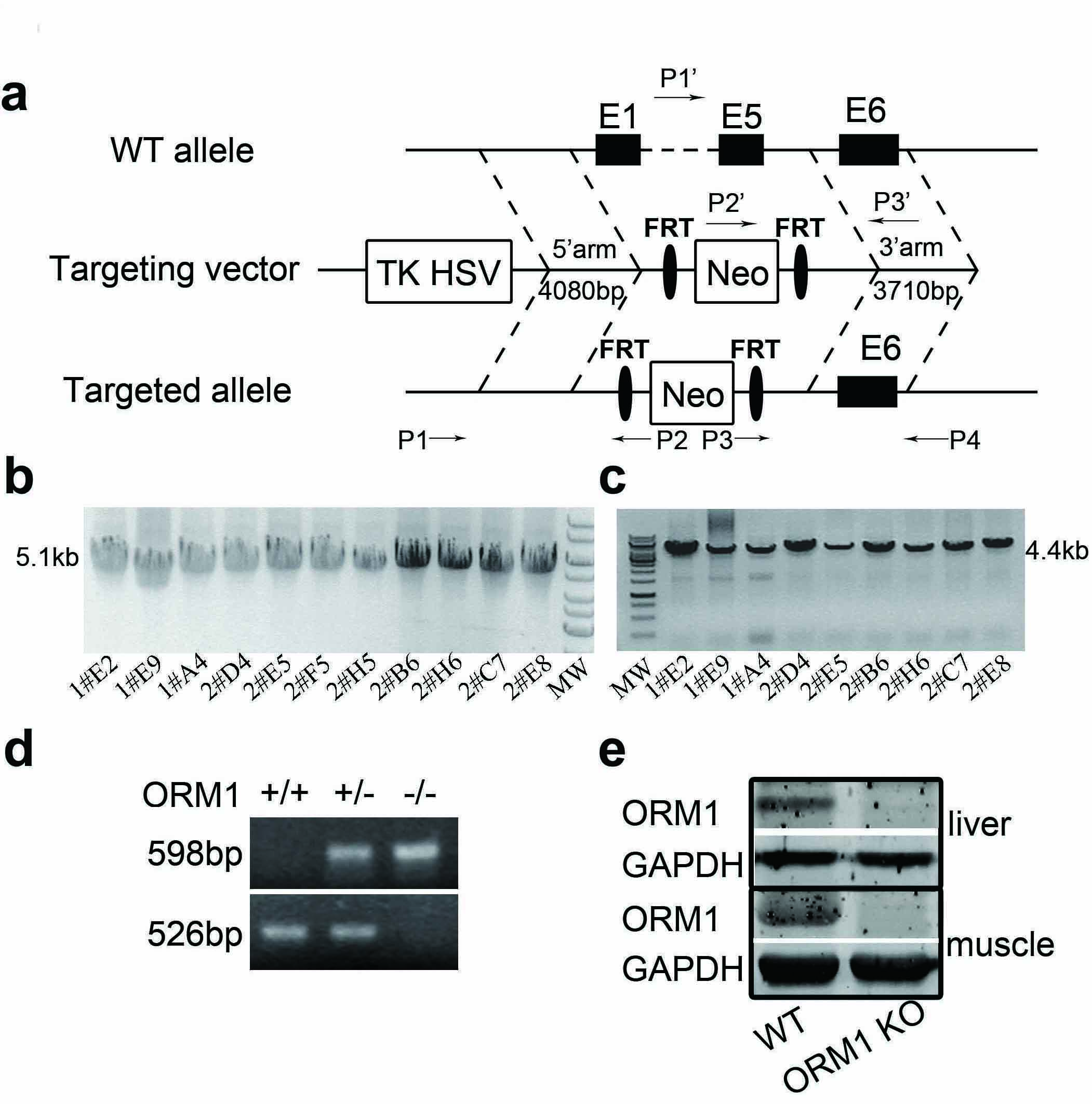
**

**Fig.S6.** Generation and identification of ORM1 knockout mice. (**a**) Genomic locus, targeting vector and predicted targeting locus. (**b**) Agarose gel analysis of 5’ PCR products from positive ES clone’s genomic DNA. (**c**) Agarose gel analysis of 3’ PCR products from positive ES clone’s genomic DNA. (**d**) Genotyping of ORM1 knockout mice. (**e**) Representative western blots of ORM1 in liver and muscle tissues from wide type (WT) or ORM1 knockout (KO) mice.


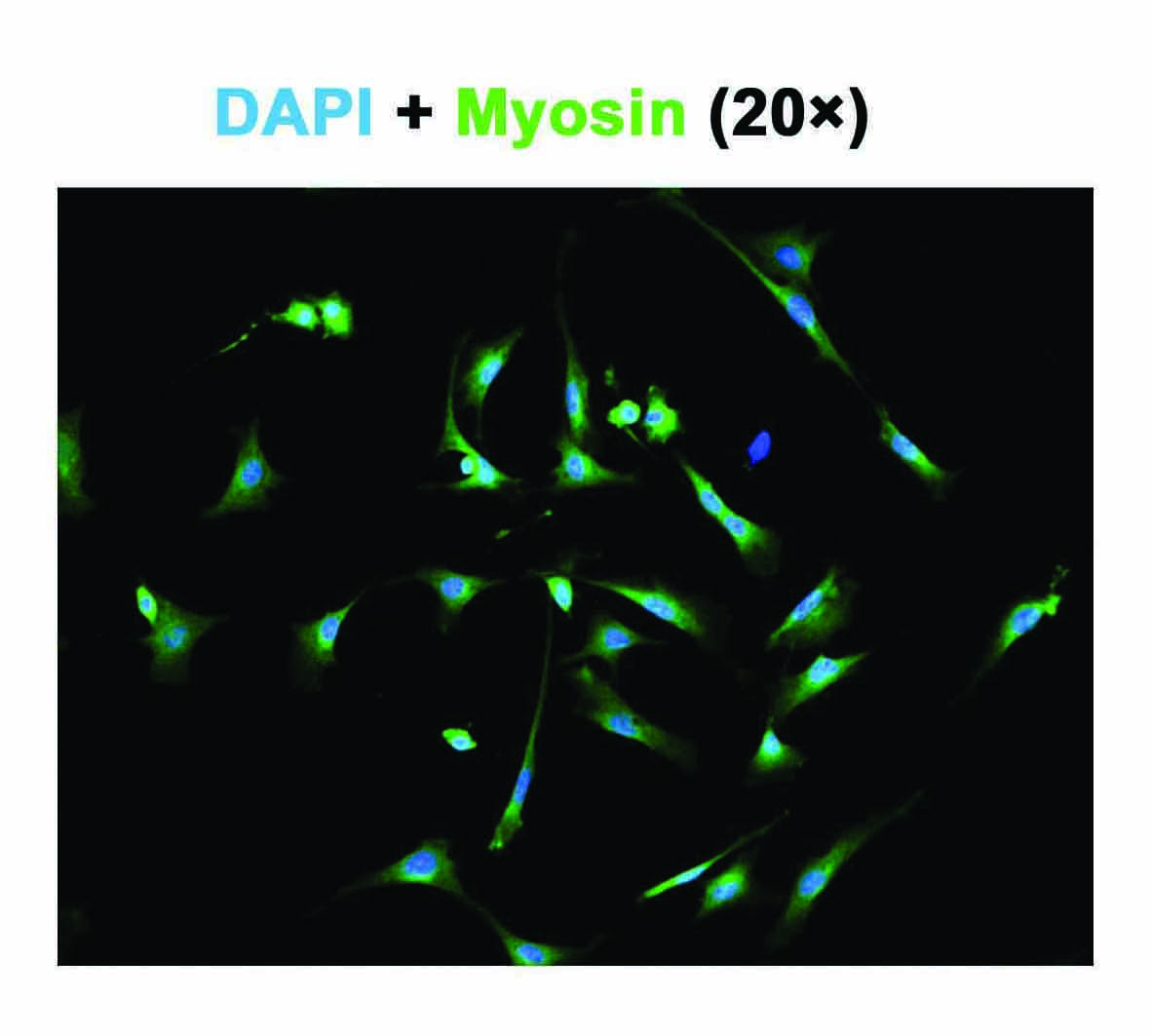


**Fig.S7**. Identification of the mouse primary skeletal muscle cells. Representative immunofluorescence image of mouse primary skeletal muscle cells stained with the specific myosin marker. Nuclei were stained with DAPI (blue).

**
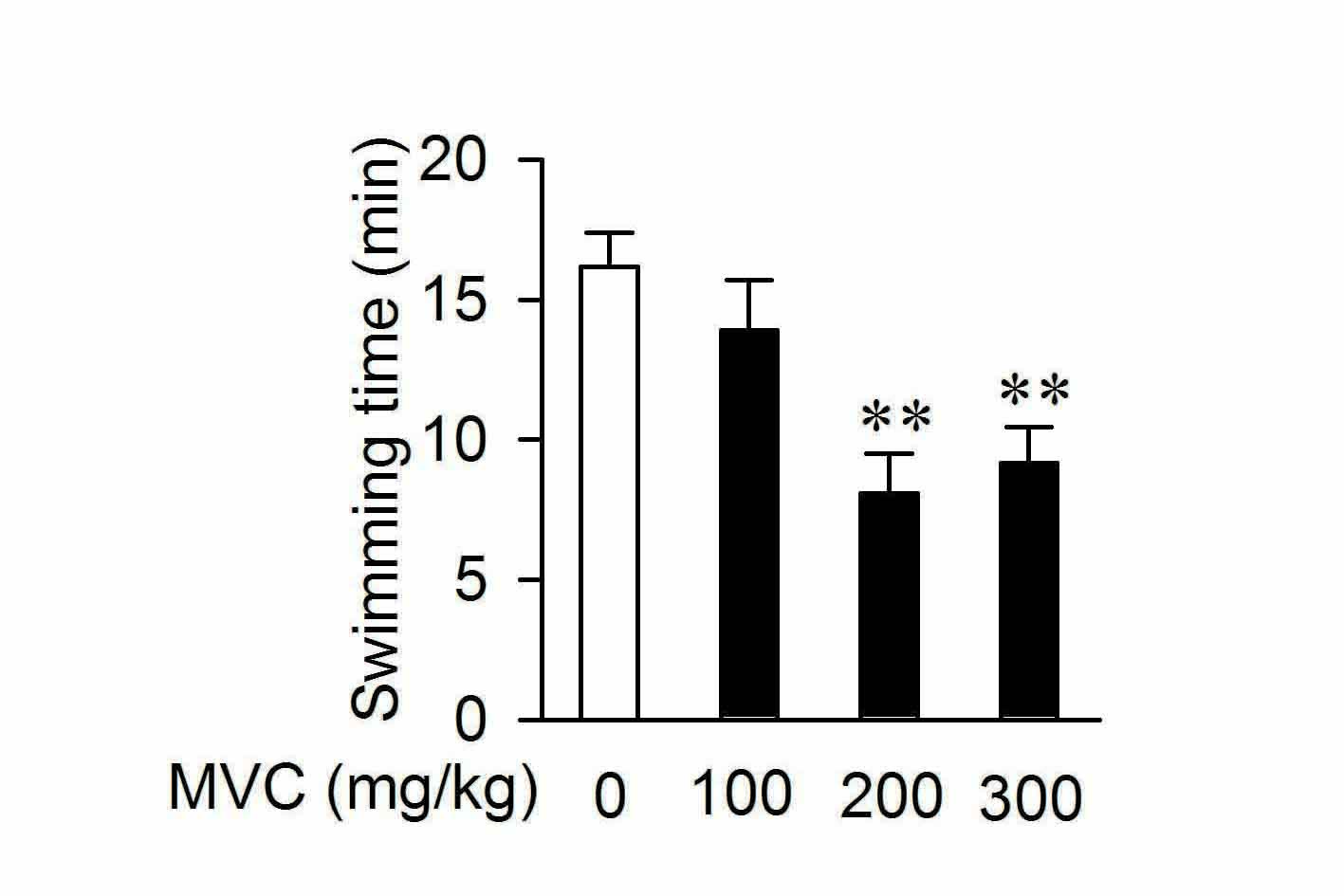
**

**Fig. S8.** Maraviroc affected swimming time in mice. Swimming test was carried out at 30 min after treatment with vehicle or Maraviroc (MVC). Maraviroc was given via gastric gavage for three days (n=7 per group). Data are mean ± s.d. ** P< 0.01 by Dunnett’s test.

**Table.S1.** Differentially Expressed Proteins in Liver from Sleep Deprived Rat

| Protein name | Spot NO. | Ratio | Access NO. | Score | Peptide matched | Coverage | MW(kd) | PI |
| --- | --- | --- | --- | --- | --- | --- | --- | --- |
| Orosomucoid 1 | 612 | +5.2 | **gi|16757980** | 95 | 7 | 20% | 23.56 | 5.64 |
| Riok 1 protein | 488 | +3.2 | **gi|50927001** | 57 | 14 | 22% | 64.558 | 5.97 |
| Quinoiddehydropteridinereductase | 859 | -2.5 | **gi|11693160** | 231 | 20 | 63% | 25.536 | 7.67 |
| Transthyretin | 618 | -3.4 |  | 141 | 7 | 34% | 13.065 | 6.04 |
| Guanidioacetatemethytransferase | 719 | -2.2 | **gi|6978873** | 265 | 21 | 58% | 26.39 | 5.69 |
| Glutathione transferase omega-1 | 796 | -2.9 | **gi|12585231** | 261 | 20 | 48% | 27.651 | 6.25 |
| Hypothetical LOC363016 | 583 | -4.9 | **gi|62079139** | 179 | 13 | 21% | 34.97 | 6.16 |
